# Effect of Paying for Performance on Utilisation, Quality, and User Costs of Health Services in Tanzania: A Controlled Before and After Study

**DOI:** 10.1371/journal.pone.0135013

**Published:** 2015-08-28

**Authors:** Peter Binyaruka, Edith Patouillard, Timothy Powell-Jackson, Giulia Greco, Ottar Maestad, Josephine Borghi

**Affiliations:** 1 Ifakara Health Institute, Plot 463, Dar es Salaam, Tanzania; 2 London School of Hygiene& Tropical Medicine, London, United Kingdom; 3 Chr. Michelsens Institutt, Jekteviksbakken 31, Bergen, Norway; Duke University Medical Center, UNITED STATES

## Abstract

**Background:**

Despite widespread implementation across Africa, there is limited evidence of the effect of payment for performance (P4P) schemes in low income countries on the coverage of quality services and affordability, consistent with universal health coverage objectives. We examined the effect of a government P4P scheme on utilisation, quality, and user costs of health services in Tanzania.

**Methods:**

We evaluated the effects of a P4P scheme on utilisation of all maternal and child immunization services targeted by the scheme, and non-targeted general outpatient service use. We also evaluated effects on patient satisfaction with care and clinical content of antenatal care, and user costs. The evaluation was done in 150 facilities across all 7 intervention districts and 4 comparison districts with two rounds of data collection over 13-months in January 2012 and February 2013. We sampled 3000 households of women who had delivered in the 12 months prior to interview; 1500 patients attending health facilities for targeted and non-targeted services at each round of data collection. Difference-in-difference regression analysis was employed.

**Findings:**

We estimated a significant positive effect on two out of eight targeted indicators. There was an 8.2% (95% CI: 3.6% to 12.8%) increase in coverage of institutional deliveries among women in the intervention area, and a 10.3% (95% CI: 4.4% to 16.1%) increase in the provision of anti-malarials during pregnancy. Use of non-targeted services reduced at dispensaries by 57.5 visits per month among children under five (95% CI: -110.2 to -4.9) and by 90.8 visits per month for those aged over five (95% CI: -156.5 to -25.2). There was no evidence of an effect of P4P on patient experience of care for targeted services. There was a 0.05 (95% CI: 0.01 to 0.10) increase in the patient satisfaction score for non-targeted services. P4P was associated with a 5.0% reduction in those paying out of pocket for deliveries (95% CI: -9.3% to -0.7%) but there was no evidence of an effect on the average amount paid.

**Conclusion:**

This study adds to the very limited evidence on the effects of P4P at scale and highlights the potential risks of such schemes in relation to non-targeted service use. Further consideration of the design of P4P schemes is required to enhance progress towards universal health coverage, and close monitoring of effects on non-targeted services and user costs should be encouraged.

## Introduction

Payment for performance (P4P) is widely regarded as a promising strategy to increase coverage and quality of maternal and child health services in low income settings and make progress towards the Millennium Development Goals 4 and 5 [1]. In 2013, 31 low and middle income countries globally were implementing P4P schemes targeting maternal and child health services[2].

P4P typically involves the allocation of funds to health facilities and to health workers based on the achievement of performance targets related to service utilisation and quality of care [3]. It is expected that health workers will respond to financial incentives by being more motivated to deliver quality care and attract patients to the facility [4–6]. The additional funding provided by P4P is also expected to improve resource availability at health facilities, enhancing the quality of services. Further, health care providers may conceivably reduce the user cost of services in a bid to achieve targets. It is also possible that by focusing on targeted services, health workers are diverted from non-targeted services resulting in their reduced quality and coverage [7].

Despite the widespread implementation of P4P across the African continent, the evidence base on P4P effects in low income settings is very limited [8–11]. There has to date been very few rigorous evaluation studies in Africa [12–14]. A study in Rwanda reported effects on a range of utilisation and quality of care outcomes targeted by the P4P scheme [12]. Services not targeted by the scheme were not examined. Studies in Burundi, examined the effect on a sub set of targeted outcomes, but also included components of care that were not directly incentivized [13–14].

Universal health coverage (UHC)–or access to care of sufficient quality, without incurring financial hardship—is now seen as an overarching goal post-2015 [15], and monitoring progress at country level is being encouraged [16]. Equity is also seen to be a critical component of UHC [17]. In order to make progress towards UHC, it is important to consider the effect of interventions such as P4P on UHC goals. By improving service coverage and quality and reducing user costs, P4P could potentially enhance progress towards universal health coverage [18, 19]. However, this needs to be balanced against the risk of reduced coverage and quality of non-targeted services which could compromise UHC.

To date, there has been limited assessment in low income settings of P4P effects on non-targeted services [20], user costs [21], or quality in terms of patient satisfaction and/or content of care [10, 13, 14, 22] and equity [13, 14, 23]. In order to assess whether P4P is compatible with universal coverage objectives we assessed the effect of a P4P scheme implemented by the Ministry of Health and Social Welfare at scale in one region of Tanzania on quality and utilisation of targeted and non-targeted services, user costs and equity by means of a prospective controlled before and after study.

## Materials and Methods

### The P4P programme in Tanzania

A payment for performance scheme was introduced in 2011 by the Ministry of Health and Social Welfare in Pwani region of Tanzania with an estimated population of just over 1 million, to inform a national P4P programme[24]. The scheme is ongoing, providing financial payments to health facilities and district and regional health managers as a bonus based on achievement of targets relating to maternal and child health care (Table 1). These payments are additional to the funding facilities receive to cover operational costs and the salaries of health workers. The targets are either for specific services (e.g. institutional delivery; postnatal care within 7 days of delivery) or for care provided during a service (e.g. two doses of Intermittent presumptive treatment (IPT) for malaria during antenatal care (ANC)). Performance targets are assessed and payment made every six months. Targets relating to partogram completion, maternal and neonatal death audits and timely submission of Health Management Information System (HMIS) reports were also introduced. Performance is measured through the HMIS which was updated to include the P4P targets. Facilities capture HMIS data on paper as before (using patient registers and monthly tally sheets) but districts started using a computerised system to enter, aggregate and analyse these data.

**Table 1:** Service indicators and performance targets for facilities.

Coverage indicators	Method	Baseline coverage (previous cycle)				
		0–20%	21–40%	41–70%	71–85%	85%+*
Institutional delivery rate	*Percentage point increase*	15%	10%	5%	5%	Maintain
% of mothers attending a facility within 7 days of delivery.	*Percentage point increase*	15%	10%	5%	5%	Maintain
% of women using long term contraceptives	*Percentage point increase*	20%	15%	10%	Maintain above 71%	Maintain
% children under 1 year received measles vaccine	*Overall result*	50%	65%	75%	80%+*	Maintain
% children under 1 year received Penta 3	*Overall result*	50%	65%	75%	80%+	Maintain
Content of care indicators						
% ANC clients on IPT2	*Overall result*	80%	80%	80%	80%+	Maintain above 80%
% HIV+ ANC clients on ART	*Overall result*	40%	60%	75%	75%+	Maintain
Polio vaccine (OPV0) at birth	*Overall result*	60%	75%	80%	80%+	Maintain

*80+: 80% or more.

Source: The United Republic of Tanzania, Ministry of Health and Social Welfare. 2011. The Coast Region Pay for Performance (P4P) Pilot: Design Document.

The programme design stipulates that at least 75% of bonus payments are distributed among health workers with the remainder being retained by the facility for investment in drugs, supplies or minor renovation. Payments are made if at least 75% of the target is achieved. Full payment is made if 100% of the target is achieved; otherwise 50% of the potential payout is made. The maximum payout per cycle is USD 820 for dispensaries; USD 3,220 for health centres; and USD 6,790 for hospitals. The health worker component equates to about 10% of their monthly salary.

Facility performance data are verified each cycle by national, regional and district stakeholders by comparing aggregate data to facility registers. District and regional managers receive bonus payments of up to USD 3,000 per cycle based on the performance of facilities in their district or region.

To participate in the scheme facilities must open bank accounts. The National Health Insurance Fund is the fund holder and disburses funds to these accounts based on performance in each cycle.

### Evaluation design

The evaluation study received ethical approval from the Ifakara Health Institute institutional review board and the ethics committee of the London School of Hygiene & Tropical Medicine. The study protocol has been previously reported[25].

**Fig 1:**
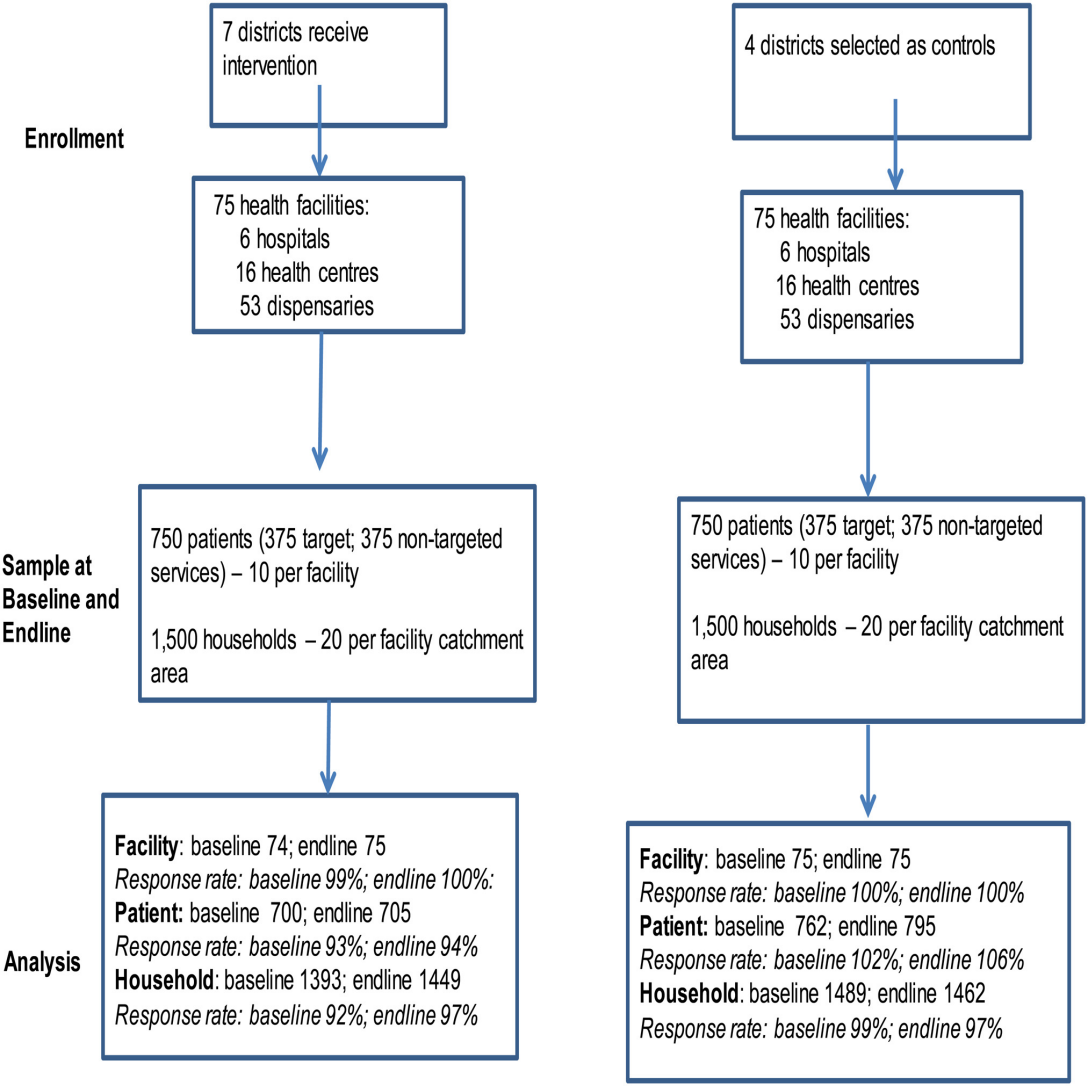
Study Profile.

A controlled before and after study design was employed. Surveys were conducted in January 2012 and 13 months later. The intervention was implemented across an entire region. However, core implementation occurs at the health facility level and eligibility to participate in the scheme is also determined at this level. Therefore, the health facility was the primary sampling unit. Intervention facilities were sampled from all seven intervention districts. Facilities were sampled from those that were eligible to participate in the payment for performance scheme (they offered reproductive and child health services and had submitted HMIS data for the previous year, enabling performance targets to be set). All eligible hospitals (n = 6) and health centres (n = 16) from the intervention districts were included in the sample along with all eligible faith-based and parastatal dispensaries (n = 11). Public dispensaries were sampled at random with probability proportional to the number of public dispensaries in a given district (n = 42). The same number of facilities were sampled from four neighbouring comparison districts (Kilwa, Mvomero, Morogoro town and Morogoro rural) which were similar to intervention districts in relation to poverty and literacy rates, the rate of institutional deliveries, infant mortality, population per health facility and the number of children under one year of age per capita. Comparison facilities were selected based on their similarity to intervention facilities in relation to annual outpatient care visits and staffing levels. A total of 150 facilities were sampled, 75 facilities in intervention and comparison sites respectively (Fig 1). In Pwani region, 46% of all eligible facilities were included in the sample and 34% of all facilities in the comparison districts.

Primary data were collected from three sources. We conducted a survey of households of women who delivered in the previous 12 months from the catchment area around the sampled facilities to determine population coverage of targeted maternal and child health services, satisfaction with delivery care, provider kindness during delivery and user costs for three of the targeted services all of which should be free at government facilities, although user fee exemptions are not well enforced [26]. Data on socio-economic characteristics were also compiled. A total of 3,000 households were sampled at each round, sufficient to detect an 11 percentage point increase in institutional deliveries (from 50 to 61%), with an assumed coefficient of variation (standard deviation/mean) of the true rates between clusters within each group, ‘k’ value of 0·25, 90% power, and a 5% significance level, assuming a 90% response rate [27].

We conducted exit interviews with patients attending antenatal or postnatal care, or women with children under-one year of age coming for a preventive check up or an immunisation to assess adherence to national clinical guidelines for ANC, waiting and consultation time and patient satisfaction with inter-personal care for targeted and non-targeted services. A total of 1,500 patients were sampled at each round, sufficient to detect a 17% reduction in waiting time from 114 minutes (SD 66) to 95 minutes, with a k value of 0·25, 80% power and a 5% significance level (Fig 1).

Finally, a survey was conducted at each facility to gather monthly service utilisation data for targeted and non-targeted services directly from patient registers for the period January 2010 to December 2012.

Baseline surveys were conducted in January 2012, after health workers had received training on P4P which took place during the second half of 2011. The risk here is that the programme may have affected outcomes at baseline, limiting observable effects. We addressed this issue within the household survey by sampling women who had delivered in the 12 months before the training of health workers started (October 2011) and considered trends since the start of 2010 in the analysis of patient register data from facilities. It was not possible to make such adjustments to the exit interview data. However, baseline outcomes from the exit survey were similar between intervention and comparison sites. A few years prior to the start of the P4P scheme, the government launched a national P4P programme, but this was never fully implemented. Health workers were therefore unlikely to have been convinced by the scheme and changed their behavior in response to it until the first payment was made, an assumption supported by formative research prior to the baseline.

### Outcome measures

As a complex health systems intervention, we sought to capture the effects of P4P on a broad set of pre-specified indicators, relating to service coverage, quality and user cost, and their distribution across socio-economic groups (S1 Table). Service use was measured for all targeted services shown in Table 1 and non-targeted services (outpatient department (OPD) services) as well as for a set of services closely related to targeted services (at least one ANC consultation, four or more ANC visits, postnatal care (PNC) within 2 months of delivery) to check for ‘spillover’ effects [25]. Service quality was measured in relation to content of care for ANC through a 21 item index based on clinical guidelines. We generated an index based on the arithmetic mean score across all item. Patients’ experience of quality was measured for targeted and non-targeted services by waiting time (in minutes), kindness during delivery (using a 10 point scale) and patient satisfaction with provider-client interactions (using an index of 13–19 items adapted from the World Bank, Impact Evaluation Toolkit) [28]. Adherence to exemption policy (free care) was measured as the share of patients paying out of pocket or giving gifts to service providers. We also measured the average amount paid for ANC, delivery care and PNC. Distributional effects were assessed by comparing outcomes across wealth terciles, for any statistically significant utilisation, quality or cost outcome. We generated a wealth index based on ownership of assets and housing particulars using principal components analysis, following the methods outlined in [29, 30]. We ranked individuals according to their index score and generated wealth terciles, three equally sized groups. We measured the effect of P4P among the poorest relative to the least poor tercile, and among the middle tercile relative to the least poor tercile.

### Statistical analysis

We identify the effects of P4P by comparing changes in outcomes in facilities with P4P to changes in outcomes in facilities without P4P. A difference-in-difference regression analysis was used to assess the effect of P4P on outcomes with facility fixed-effects, as shown in Eq 1.

$$Y_{ijt}\;=\;\beta_{0}+\beta_{1}(P4P_{j}\;\times \;\delta_{t})\;+\;\beta_{2}\delta_{t}\;+\;\beta_{3}X_{ijt}\;+\;\gamma_{j}\;+\;\varepsilon_{ijt}$$ 1

We used ordinary least squares (OLS) with standard errors clustered at the facility level, or the facility catchment area, as this is the level at which sampling was done and the intervention is directed at facilities. We confirmed the robustness of our results to using non-linear (logit) models for binary outcomes, and clustering standard errors at the district level, accounting for the small number of clusters using the bootstrapping method proposed by Cameron et al [31]. In all models, we included facility fixed effects (γ_*j*_)to control for facility-level unobserved time invariant characteristics and year fixed effects (*δ*_*t*_) a dummy variable taking the value of 0 at baseline and 1 at endline. We also control for individual-level characteristics (education, religion, marital status, occupation, age, number of pregnancies) and household characteristics (insurance status, number of household members, household head education, and wealth based on ownership of household assets and housing particulars) that are known to affect the outcomes (*X*_*ijt*_). The effect of P4P on outcomes is estimated as *β*_1_. To assess whether P4P has differential effects by socio-economic status, we ran extended models, by interacting the P4P variable with the time dummy and the household wealth terciles. The ability of the difference-in-difference approach to accurately identify the causal effect of P4P, relies on the assumption that trends in outcomes between intervention and comparison sites were running parallel prior to the start of the intervention, or that the comparison site represents a valid counterfactual, or measure of what would have happened in intervention facilities without the intervention. While this assumption can never be formally tested, we verified that trends in a number of outcomes were similar between the intervention and comparison areas prior to the introduction of P4P (S1 File). The analysis of facility register data is restricted to facilities with 30 months or more data (S2 File). All statistical analyses were done with STATA (version 12).

### Ethics

The evaluation study received ethical approval from the Ifakara Health Institute institutional review board (approval number: 1BI1IRB/38) and the ethics committee of the London School of Hygiene & Tropical Medicine. Study participants provided written consent to participate in this study, requiring them to sign a written consent form that was read out to them by the interviewers. This consent form was reviewed and approved by the ethics committees prior to the start of the research.

## Results

The response rate for each of the surveys varied from 92% to 100%, with the lowest rate being in the household survey in the intervention area at baseline (Fig 1). The characteristics of women, patients and facilities across intervention and comparison sites were generally similar (S3 File). Women from intervention areas were more likely to be Muslim, married, and poor than their counterparts in comparison areas (Tables C and D in S3 File). They were less likely to do farming and have secondary education. These differences are not a problem for the difference-in-difference analysis which controls for any baseline differences between groups. The key assumption that trends in a number of key outcomes prior to the introduction of P4P were statistically similar between intervention and comparison groups was confirmed (S1 File).

Almost all of the intervention facilities sampled (96%) had received some bonus payments during the 13 month evaluation period. The funds that were retained at the facility level were generally used to purchase drugs and supplies. Table 2 reports our estimates of the impact of P4P on service use. We found a significant increase in two of the eight targeted indicators: a 10.3% increase (95% CI: 4.4% to 16.1%) in the share of women receiving two doses of IPT during ANC; an 8.2% increase (95% CI: 3.6% to 12.8%) in the share of women having an institutional delivery. There was a positive effect on polio immunization at birth, with coverage increasing by 5.6% (95% CI: -1.0% to 12.2%), but this was not significant at p<0.05 level. There was a greater increase in institutional deliveries among the middle tercile relative to the least poor tercile, and among the poorest tercile relative to the least poor tercile for deliveries in public facilities; but the effect was only significant at the p<0.1 level (S2 Table). When standard errors were clustered at the district level, the effect on institutional deliveries was only significant a p<0.1 level and there were no differential effects by socio-economic group (Tables A and E in S4 File).

**Table 2:** Direct and indirect effect of P4P on the use of targeted services.

	Baseline survey				Difference in difference, effect			
Outcome Variables	Intervention	Comparison	Difference	P-value	N	Beta† (95% CI)	P-Value	%D*
**Targeted services**								
At least 2 doses of IPT during ANC (%)	49·5	56·7	-7·2	0·005	4759	10.3 [4.4 to 16.1]	0·001	20·8
HIV treatment during ANC (%)	7·8	6·8	1·0	0·527	5666	-0·3 [-4.2 to 3.7]	0·893	-3·8
Institutional delivery rate (%)	84·7	86·8	-2·1	0·350	5747	8.2 [3.6 to 12.8]	0·001	9·7
Institutional delivery rate (%)(public)	76·8	77·8	-1·0	0·786	5747	6.5 [1.3 to 11.7]	0·015	8·5
Polio vaccine at birth (%)	77·4	78·5	-1·1	0·668	5747	5.6 [-1.0 to 12.2]	0·093	7·2
Measles (%)	51·4	53·3	-1·9	0·654	1252	9.6 [-2.5 to 21.6]	0·119	18.7
Penta 3 doses^ (%)	76·4	79·9	-3·5	0·243	2574	2.4 [-6.6 to 11.4]	0·597	3·1
Postnatal care in facility<7 days (%)	21·5	16·9	4·6	0·043	5745	0·6 [-5.0 to 6.3]	0·823	2·8
Use of any family planning (%)	36·7	39·2	-2·5	0·398	5514	-0·7[-7.4 to 6.0]	0·844	-1.9
**Non-targeted aspects of targeted services**								
Any ANC visit(%)	97·2	99·9	-2·7	0·001	5742	3.3 [1.5 to 5.1]	0·000	3·4
Four or more ANC visits (%)	65·0	71·2	-6·2	0·020	5674	3.9 [-2.7to 10.4]	0·245	6·0
Postnatal care in facility < 2 months (%)	27·7	23·4	4·3	0·120	5745	-1.6 [-8.1 to 4.9]	0·625	-5·8

*The % D = (beta / baseline mean) × 100, where the baseline mean of the dependent variable is for the intervention group.

^†^The Beta is the estimated intervention effect controlling for a year dummy, facility-fixed effects, individual-level and household characteristics.

^^^Among infants aged 6–11 months.

For services closely related to the targets, there were positive effects on overall coverage for ANC increasing by 3.3% (95% CI: 1.5% to 5.1%), however, there was no evidence of an effect on the proportion of women having four or more ANC visits, or postnatal care in the previous two months (Table 2). These results were consistent when standard errors were clustered at the district level (Table A in S4 File) and in the nonlinear model (Table A in S5 File).

There was no effect on the use of non-targeted services proxied by total outpatients visits for those under and over five years of age when considering all facilities (the effect on outpatient visits for those aged under five years was significant when clustering at the district level (Table B in S4 File)). However, the use of these services decreased significantly in lower level facilities (in dispensaries) by 57.5 visits per month for children under five years of age (a reduction of 35% compared to baseline levels) (95% CI: -110.2 to -4.9). The decrease was by 90.8 visits per month (a reduction of 33% compared to baseline levels of utilisation) for those aged over five (95% CI: -156.5 to -25.2) (Table 3). The results were consistent when standard errors were clustered at the district level (Table B in S4 File).

**Table 3:** Effect of P4P on the use of non-targeted services.

	Baseline survey					Difference in difference, effect			
Outcome Variables	Intervention	Comparison	Difference	P-value	Facilities	N	Beta† [95% CI]	P-Value	%D*
Outpatient visits per month > 5 yrs	359·5	287·3	72·2	<0·001	96	3353	-15·8 [-101·1 to 69·5]	0·714	-4·4%
Outpatient visits per month > 5 yrs, dispensaries	276·8	235·4	41·4	0·006	69	2538	-90·8 [-156·5 to -25·2]	0·007	-32·8%
Outpatient visits per month < 5 yrs	223·9	193·7	30·2	0·011	93	3247	-41·1 [-93·2 to 10·9]	0·120	-18·4%
Outpatient visits per month < 5 yrs, dispensaries	164·8	172·6	-7·8	0·441	72	2428	-57·5 [-110·2 to -4·9]	0·033	-34·9%

Note to Table: N = facility months;

*The % D = (beta / baseline mean) × 100, where the baseline mean of the dependent variable is for the intervention group.

^†^The Beta is the estimated intervention effect controlling for a year dummy and facility-fixed effects.

There was no effect of P4P on measures of patient experience of care for targeted services. There was an increase in provider kindness reported by patients during delivery, a positive 0.38 point increase in the mean kindness score (95% CI: -0.06 to 0.80), although this was not significant at p<0.05 level (Table 4). There was an increase in patient satisfaction with inter-personal care for non-targeted services with a 0.05 increase in the mean score (7.2% increase relative to baseline) (95% CI: 0.01 to 0.10). There was no differential effect on quality of care indicators by socio-economic group. The results were consistent when standard errors were clustered at the district level (Table C in S4 File).

**Table 4:** Effect of P4P on quality of care.

	Baseline survey				Difference-in-difference, effect			
Outcome Variables	Interventionmean [sd]	Comparisonmean [sd]	Difference	P-value	N	Beta† [95% CI]	P-Value	%D*
**Targeted services**								
ANC content of care index	0·53 [0·19]	0·49 [0·17]	0·04	0·103	680	-0·06 [-0·13 to 0·02]	0·118	-11·3
Index of patient satisfaction with interpersonal care for outpatient services	0·72 [0·16]	0·70 [0·17]	0·02	0·426	1247	0·04 [-0·01 to 0·09]	0·138	5·6
Index of patient satisfaction with interpersonal care during deliveries^	0·63 [0·19]	0·64 [0·18]	-0·01	0·411	4941	0·01 [-0·02 to 0·04]	0·505	1·6
Patient assessment of staff kindness during delivery score (1–10)^	7·2 [2·7]	7·6 [2·7]	-0·4	0·009	4920	0·38 [-0·06 to 0·82]	0·088	5·3
Waiting time in minutes	50·9 [56·8]	48·8 [61·2]	2·1	0·793	1211	5·5 [-17·4 to 28·4]	0·636	10·8
Consultation time in minutes	15·8 [12·5]	13·6 [10·3]	2·2	0·117	1211	-0·8 [-4·4 to 2·8]	0·650	-5·1
**Non-targeted services**								
Index of patient satisfaction with interpersonal care	0·69 [0·18]	0·74 [0·15]	-0·05	0·007	1170	0·05 [0·01 to 0·10]	0·030	7·2
Waiting time in minutes	51·4[57·4]	43·7 [45·6]	7·7	0·213	1126	-10·3 [-29·7 to 8·9]	0·292	-20.0
Consultation time in minutes	13·9 [11·5]	13·7 [12·2]	0·2	0·899	1130	-0·3 [-3·3 to 2·7]	0·868	-2·2

Note to Table: Same sizes as indicated at top except where indicated ^.

^^^ Data from household survey: sample size;

*The % D = (beta / baseline mean) × 100, where the baseline mean of the dependent variable is for the intervention group.

^†^The Beta is the estimated intervention effect controlling for a year dummy, facility-fixed effects, individual-level and household characteristics

P4P resulted in a greater enforcement of exemptions for delivery care at public facilities (a 5.0% reduction in those paying out of pocket (95% CI: -9.3% to -0.7%) (Table 5). The effect was consistent when standard errors were clustered at the district level (Table D in S4 File) and in the nonlinear model (Table B in S5 File). However, there was no effect on exemptions for ANC or PNC, nor on the average amount paid or on the provision of gifts by patients for these services in the linear model. A negative effect was identified on the likelihood of paying out of pocket for antenatal care, and a positive effect on giving a gift in the non-linear model but this was only significant at p<0.1 level (Table B in S5 File). There was a greater reduction in the probability of paying for deliveries among the poorest tercile relative to the least poor tercile (S2 Table), but the effect was only significant at p<0.1 level, and the effect was not present when standard errors were clustered at the district level and in the non-linear model (S4 and S5 Files). There were no other differential effects on cost indicators by socio-economic group.

**Table 5:** Effect of P4P on the cost of services in public facilities.

	Baseline survey				Difference in difference, effect			
Outcome Variables	Intervention	Comparison	Differ-ence	P-value	N	Beta† [95% CI]	P-Value	%D*
Prob. of paying fpr ANC (%)	8.1	7.5	0.6	0·711	5091	-2.7 [-6.0 to 0·6]	0·110	-33·3
Prob. of paying for delivery care (%)	16.5	11.9	4.6	0·026	4485	-5.0 [-9.3 to -0·7]	0·023	-30·3
Prob. of paying for PNC (%)	6·0	7·6	-1·6	0·421	1257	2.1 [-3.7 to 7.9]	0·476	35·0
Amount paid for ANC, mean USD[sd]	0·23 [1·5]	0·15[1·0]	0·08	0·201	5091	0·12 [-0·11 to 0·35]	0·310	52·2
Amount paid for delivery, mean USD [sd]	1·80 [8·7]	2·18 [16·5]	-0·38	0·509	4485	0·19 [-1·17 to 1·55]	0·780	10·6
Amount paid for PNC, mean USD[sd]	0·34 [1·9]	0·96 [6·8]	-0·62	0·119	1257	0·43 [-0·23 to 1·08]	0·202	126·5
Provided a gift for ANC (%)	1·7	1·2	0·5	0·403	5126	1.2 [-0·5 to 2.8]	0·158	70·7
Provided a gift for delivery (%)	17·4	18·8	-1·4	0·586	4499	-2.8[-8.2 to 2.6]	0·302	-16·7
Provided a gift for PNC (%)	7·6	4·5	3·1	0·134	1283	-0·4 [-6.4 to 5.6]	0·897	-6·6
Value of gift for ANC, mean USD [sd]	0·05 [0·5]	0·03 [0·3]	0·02	0·177	5126	-0·04 [-0·17 to 0·09]	0·569	-80·0
Value of gift for delivery, mean USD [sd]	0·61 [2·1]	0·59[1·5]	0·02	0·875	4499	-0·21 [-0·46 to 0·04]	0·100	-34·4
Value of gift for PNC, mean USD [sd]	0·33 [1·7]	0·14 [0·8]	0·19	0·069	1283	0·004 [-0·32 to 0·33]	0·978	1·2

*The % D = (beta / baseline mean) × 100, where the baseline mean of the dependent variable is for the intervention group.

^†^The Beta is the estimated intervention effect controlling for a year dummy, facility-fixed effects, individual-level and household characteristics

## Discussion

We report on the effect of P4P in relation to a broad set of outcomes—by considering effects on non-targeted as well as targeted services, service quality, user costs and equity. The evaluation of the P4P scheme in Tanzania revealed mixed findings in relation to service coverage. There were significant positive effects for two out of eight performance indicators: coverage of institutional deliveries and provision of two doses of anti-malarials during pregnancy. While no overall effect on the use of non-targeted services was found, there was a reduction in the use of non-targeted services at dispensaries which represent the majority of facilities in the region. With regards to quality, there was no improvement in antenatal content of care and there was no effect on patient satisfaction with inter-personal care for targeted services, but there was a significant improvement in patient satisfaction with inter-personal care for non-targeted services. With regards to user costs, P4P led to greater enforcement of exemptions at public facilities for one out of the three targeted services considered (deliveries). However, we found no improvement in financial protection as measured by the amount paid by the patient for each service. Finally, there is an indication that the effects on the rate of institutional deliveries in public facilities may have been pro-poor. No other equity effects were identified.

The closest antecedents to our study are the evaluations of P4P in Rwanda and in Burundi. In Rwanda, the scheme led to improvements in four out of 14 targeted services [12]. Only two of the targets were the same in both countries: deliveries, where effects were of a similar magnitude despite higher baseline coverage in Tanzania [12] and IPT during ANC, where no effect was documented in Rwanda. In contrast to the current study, which showed the effect of the P4P package (resources, incentives and performance verification), the Rwanda study isolated the incentive and verification effects from the resource effect of P4P.

In Burundi, an evaluation of a pilot programme reported a 22% increase in institutional deliveries and a 10% increase in antenatal care during the first phase of implementation, and an effect on family planning (during the second phase of implementation) [13]–although only the effects on a subset of all incentivised indicators were evaluated. The effect of deliveries disappeared when evaluating the national programme, although the effect remained borderline significant when restricting to those who had been exposed to the intervention for at least a year [14]. In Burundi, like in our study, the evaluation assessed the resourcing effect of P4P as well as the incentive effect.

Ours is one of the only studies to report effects of P4P on PNC[9], but there was no evidence of an effect, possibly because cultural barriers may prevent women from seeking care so early after delivery [32, 33]. Community based PNC may be a target that is more readily achievable by providers and acceptable to communities. Other studies have reported P4P effects on family planning service availability and quality [22]. However, it is also acknowledged that achieving such effects may take longer and hence, we may have been limited in our ability to detect effects due to the short time frame of the evaluation [9].

Most studies from low income setting have assessed P4P effects on targeted services with little consideration of potential ‘spillover’ effects. The positive effect on the use of a service (ANC) directly related to a target (two doses of IPT) echoes findings from high income settings [34, 35]. The fact that no effect was detected for four or more ANC visits suggests that spillover effects are restricted to services closely related to the target. The evaluation in Burundi also documented positive effects on components of care provided during ANC which were not directly incentivized [14]. The significant reduction in outpatient visits at dispensaries in our study is consistent with concerns on the deterioration of non-targeted services reported elsewhere [35, 36]. The result is also supported by findings from a costing study we conducted which estimated that health workers spent 17% of their time each month on data generation and verification activities related to P4P at primary level facilities including dispensaries, reducing available time to attend to patients [37]. Given these mixed findings, the net effect of P4P on service utilisation is unclear.

Relatively few studies of P4P in low income settings have reported effects on service quality. Similar to our study, no effects on patient satisfaction with care were identified in Burundi, although improved satisfaction and content of care were reported in the Democratic Republic of Congo [38]. No previous studies have considered provider kindness during deliveries, although there is growing recognition of the importance of respect and dignity during delivery [39]. The lack of effect on overall adherence to clinical guidelines or on patient-provider interactions for antenatal care is perfectly plausible, as targets in Tanzania emphasized service coverage rather than quality. The increased patient satisfaction with interpersonal care for non-targeted services is hard to explain but may be due to the fact that there were relatively fewer patients attending these services.

The reduction in the probability of paying out of pocket for delivery care in our study is consistent with a study in China [40]. In the Democratic Republic of Congo, a 45 percent increase in overall out of pocket health spending among households was associated with a P4P scheme that gave freedom to providers to adjust user fees [21]. We were unable to assess the effect of P4P on the affordability of care, and incidence of catastrophic payments, which are important dimensions of financial protection [41], this would be an important area for future research.

This study identified a potential pro-poor effect of P4P on deliveries at public health facilities and the probability of paying for delivery care, although these effects were not consistent across different model specifications. The evaluation in Rwanda found no differential effects by socio-economic groups [23]. The Burundi pilot found no differential effect by wealth group [13], but a pro-poor effect on full immunization coverage was documented in the national evaluation, and a pro-rich effect on institutional deliveries [14]. In higher income countries, no effect of P4P has been found on inequities in age, sex and ethnicity [42, 43].

The data and study design used in this evaluation merit close scrutiny. First, measures of non-targeted service use in our study relied on patient register data which were incomplete for many facilities, limiting the available sample for analysis. Reporting of data may also be prone to ‘gaming’: modifications to the health management information system (HMIS) were introduced alongside P4P in the intervention area and facilities were paid based on the completeness and timeliness of HMIS reports. Second, conducting credible evaluations of health system interventions is challenging and in the current study there was no opportunity to randomly allocate the P4P scheme. By collecting data in comparison districts over time and verifying that pre-intervention trends were similar, we believe our study represents a rigorous attempt to identify the effects of P4P. Third, generalising findings from an evaluation of an intervention as complex as P4P is far from easy and the most promising way forward is likely to be the accumulation of evidence from multiple settings over time. Our study was large scale and the particular scheme in Tanzania bears many of the same features as P4P schemes being tested in other low- and middle- income countries. Our analysis does not adjust results for multiple outcomes, or the risk when conducting multiple significance tests that some significant results might arise by chance. We have generated indices where possible to collapse multiple outcomes into a composite outcome and minimize the number of outcomes (for example for quality of care). However, in other cases, p-values that are lower than those typically considered ‘significant’ might be given greater weight. Lastly, the evaluation was conducted following 13 months of implementation. It is hard to know when is the optimal time to measure the effects of programmes seeking to change behavior such as P4P. Evaluating effects too early may underestimate effects. However, there is also a risk of evaluating effects too late, as a recent study in the United Kingdom indicated that P4P effects were not sustained over time [44]. There is no real answer as to the optimum time to evaluate as there is limited evidence on how effects evolve over time and how long it takes to get an effect. These are important areas for future research.

There has to date been little consideration of the ‘mechanism’ of P4P effect, or how P4P affects the health system to deliver outcomes [7]. Our starting hypothesis was that utilisation would increase through improved quality and reduced service costs resulting from changed provider behaviour. Potential improved kindness during delivery and a better enforcement of delivery care exemptions are consistent with increased delivery care use. A process evaluation conducted in parallel with this study also revealed that strategies employed by providers to meet targets largely centred around the achievement of the delivery care target, for example, paying traditional birth attendants for referrals, and extending opening hours. In Burundi, it was reported that effects on deliveries operated through antenatal care, however, this does not appear to be the case in Tanzania, as the inclusion of the antenatal care variable in our delivery care outcomes model, did not mediate the effect of P4P on institutional deliveries.

The question begs as to why health workers focused their efforts on delivery care. The increase in institutional deliveries in Rwanda was hypothosised to be linked to the higher incentive level attached to this compared to other services [12]. However, in Tanzania there was no difference in the payments made by service. Achievement of the delivery care target is rewarded equally to other service targets. Health workers may nevertheless perceive deliveries to be more profitable, enabling the potential achievement of two targets at once—the delivery and polio vaccine at birth target. The achievement of the IPT target may have demanded limited effort as ANC coverage was already very high.

P4P is being widely implemented across Africa and in many other countries in Asia. While there is variation in design and implementation across countries [7], the general principle of financial incentives for targeted services is consistent. Policy makers implementing or planning to implement P4P should carefully consider the design of P4P schemes to ensure they are compatible with universal coverage goals. In order to minimize the risk of reductions in the use of non-targeted services, for example, implementers might consider regularly rotating service targets, or incentivizing overall performance across all services. Routine monitoring of effects on non-targeted services should be encouraged. Ongoing assessment of effects on costs faced by users and service affordability is also important, and P4P schemes may consider incentivizing pre-payment and pooling arrangements to enhance compatibility with UHC, or the introduction of demand side financing strategies such as vouchers, insurance or cash transfers might be considered alongside P4P [45]. Incentivising quality of care is also clearly important to ensure effective service delivery that will improve outcomes. However, reliably measuring quality and integrating routine quality checks into information systems is likely to prove challenging for many countries [8].

While P4P achieved limited effects on targeted maternal and child health services, overall progress towards universal coverage was mixed. Further evaluation research on distributional effects, effects on non-targeted service use and quality, and financial protection is urgently needed, to determine whether P4P will help achieve progress towards UHC or undermine it.

## Supporting Information

S1 File Analysis of pre-trends in household and facility survey data. (DOCX)

S2 File Analysis of facility utilisation data. (DOCX)

S3 File Comparison of baseline characteristics. (DOCX)

S4 File Results using linear model with standard errors clustered at the district level, adjusting for limited number of clusters using the wild cluster bootstrap approach outlined in Cameron et al. (2008). (DOCX)

S5 File Results using logit model on all binary outcomes (with marginal effects and standard errors clustered at the facility level). (DOCX)

S1 Table Overview of core indicators for each of the surveys. (DOCX)

S2 Table Equity effects of P4P. (DOCX)
