# Supplementary material for: Effect of Paying for Performance on Utilisation, Quality, and User Costs of Health Services in Tanzania: A Controlled Before and After Study
Source: PLoS One. 2015 Aug 28;10(8):e0135013. doi: 10.1371/journal.pone.0135013 (PMC4552688; doi:10.1371/journal.pone.0135013)
Supplement: S1 File — (DOCX) [file pone.0135013.s001.docx]

**S1 File: Analysis of pre-trends in household and facility survey data**

The difference-in-difference empirical strategy rests on the parallel trends assumption – that is, in the absence of the payment for performance (P4P) intervention trends in outcomes do not differ between intervention and comparison areas. This assumption can never be formally tested. However, we are able to provide supportive evidence by examining pre-trends – trends prior to the introduction of P4P – for those outcomes that we are able to exploit the longitudinal nature of the data.

**Outcomes in household data**

The household survey interviewed women who had given birth in the past one year and collected data on the month and year of childbirth. We are therefore able to generate longitudinal measures for outcomes related to care given at the time of childbirth. These outcomes include: proportion of women who gave birth in a health facility; proportion of women who had a caesarean section; proportion of women who breastfeed within one hour of birth; and proportion of women who had to pay for delivery care. Childbirth is a single event that occurs at a particular moment in time. The same is not true for other outcomes, such as those related to antenatal care.

We first present descriptive evidence showing that the four outcomes of interest at baseline are similar in terms of both the level and the pre-trend (Figure A). More formally, we test for a divergence in pre-trends by estimating the following regression:

$y_{ijt}=\beta t+{\gamma D}_{j}t+\theta_{j}+X_{ij}+\varepsilon_{ijt}$ (1)

where $y_{ijt}$ is the outcome of individual *i* in facility catchment *j* in month *t*, *t* represents the month since the start of the data period, $\beta$ is the monthly trend is the comparison districts, $\gamma$ is the difference in the trend in the intervention districts, $\theta_{j}$ are facility fixed-effects, $X_{ij}$ are characteristics of the woman, and $\varepsilon_{ijt}$ is the error term.

We were unable to reject the null hypothesis of equal pre-trends for any of the outcomes. The estimated values of $\gamma$ were: i) facility based delivery coefficient of -0·0001 (95% CI -0·0004, 0·0002; p value=0·580); c-section coefficient of -0·00002 (95% CI -0·0002, 0·0002; p value=0·893); early breast-feeding coefficient of 0·0001 (95% CI -0·0002, 0·0004; p value=0·683); and pay for delivery coefficient -0·0001 (95% CI -0·0001, 0·0002; p value=0·618). Taken together, these findings provide reassuring evidence that the introduction of the P4P scheme is orthogonal to the error term.

**Figure A: Differences in pre-trends**

**Outcomes in facility data**

We tested for divergent pre-trends for utilisation outcomes from patient registers in a similar manner. As above, the coefficient on the interaction between the P4P intervention variable and the linear monthly time trend shows the difference in the pre-trend and is reported here below with the corresponding p value. There is no evidence of divergent pre-trends for the majority of outcomes studied. However, the findings do suggest that trends for family planning visits and outpatient visits under five were rising in the intervention group relative to the comparison group prior to the introduction of P4P. Impact results related to these two outcomes should therefore be interpreted with particular caution. When trends are rising faster in the intervention group we are concerned with potential positive bias – eg. a spurious finding of a positive impact. For family planning visits and outpatient visits under five we found no effect. This may be a spurious finding and in fact there was a negative effect, which simply reinforces our results from the dispensary only analysis.

Normal deliveries: 0·002 (p=0·979)

Normal deliveries (excluding hospitals): -0·036 (p=0·651)

Polio: 0.576 (p=0·235)

Measles: -0.121 (p=0·454)

DPT: 0.114 (p=0·771)

Family planning visits: 1·54 (p=0·039)

ANC visits: -0·441 (p=0·297)

ANC first visits: -0·124 (p=0·435)

Outpatient visits under five: 5·474 (p=0·037)

Outpatient visits under five (dispensaries only): 2·033 (p=0·306)

Outpatient visits over five: 5·067 (p=0·227)

Outpatient visits over five (dispensaries only): -2·565 (p=0·419)
