# Supplementary material for: Effect of Paying for Performance on Utilisation, Quality, and User Costs of Health Services in Tanzania: A Controlled Before and After Study
Source: PLoS One. 2015 Aug 28;10(8):e0135013. doi: 10.1371/journal.pone.0135013 (PMC4552688; doi:10.1371/journal.pone.0135013)
Supplement: S2 File — (DOCX) [file pone.0135013.s002.docx]

**S2 File: Analysis of facility utilisation data**

**Background**

In addition to the household data, we collected information on healthcare utilisation from the patient registers of health providers – these are the raw data that are entered into the Health Management Information Systems (HMIS). Because the payment for performance (P4P) scheme affected the HMIS, there are a number of possible concerns with these data. First, modifications to the HMIS data system were introduced alongside P4P in the intervention area. Second, facilities were paid based on the completeness and timeliness of HMIS reports. Third, providers had an incentive to over-report on utilisation (‘gaming’). Consequently data based on patient registers may be prone to bias and we place the greatest weight on the findings from the household data, collected from the community lying in the catchment area of each study facility. Nevertheless, these facility level data provide an alternative data source with which to assess the effect of P4P on healthcare utilisation. In particular, they provide the basis to examine whether there were any unintended effects on non-targeted service utilisation.

**Data**

We use utilisation data collected directly from the patient registers of health facilities surveyed as part of the impact evaluation. The facility data on utilisation are monthly, covering the years 2010, 2011, and 2012 – ie. 36 months of data for each facility. Data on the following outcomes were collected: outpatient visits over five; outpatient visits under five; antenatal care visits; antenatal care first visits; normal deliveries; Penta 3 vaccination; measles vaccination; polio vaccination; and family planning visits.

There are two data issues that must be addressed. First, as is common in such settings, facility registers were sometimes incomplete resulting in missing data. We thus restricted the analysis to facilities with 30 months of data or more. While the threshold is arbitrary, we find no substantive change to the results when other thresholds are used. Second, we address the problem of outliers by trimming the sample at the 99^th^ percentile of values for each outcome.

**Statistical analysis**

We examine the effect of P4P by comparing trends in utilisation over time between intervention and control facilities for each of the outcomes. We consider an intervention start date of July 2011 for this is when training on P4P was given to the health providers. Our estimating equation is as follows:

$y_{jt}={\delta D}_{jt}+\theta_{j}+\vartheta_{t}+\varepsilon_{dt}$ (2)

where $y_{jt}$ is utilisation of facility *j* in month *t,* and $D_{jt}$ is the introduction of the P4P intervention. The specification also includes a full set of facility fixed-effects, $\theta_{j}$, which absorb facility specific time invariant determinants of utilisation, as well as month effects, $\vartheta_{t}$. The error term is denoted by $\varepsilon_{dt}$. The effect of the P4P intervention is given by $\delta$. We report confidence intervals based on standard errors that allow for heteroskedasticity of an unspecified form and that are clustered at the health facility level.

**Results**

Difference in difference estimates of impact are shown in Table A. The results indicate a positive effect of P4P on the number of normal deliveries, consistent with the findings from the household survey. We find a positive effect on antenatal care first visits but no overall effect on the number of antenatal care visits. We next turn to non-targeted outcomes. The coefficient estimate is negative for both outpatient visits among children and outpatient visits for those over five years old. When the analysis is restricted to dispensaries the negative effects becomes larger and statistically significant. The dispensary sample included in the analysis is similar to that of the overall sample (Tables A and B in S3 File).

**Table A: Effect of P4P on service utilisation (from facility survey)**

|  | N | Beta (95% CI) | P value | % change |
| --- | --- | --- | --- | --- |
| **Targeted Services** |  |  |  |  |
| Normal deliveries incl. Hospitals | 2792 | 3·3 (0·3, 6·4) | 0·033 | 35·5% |
| Normal deliveries excl. Hospitals | 2584 | 2·9 (-0·2, 6·1) | 0·067 | 38·2% |
| Polio vaccinations | 3084 | 4·4 (-5·9, 14·7) | 0·398 | 8·8% |
| Measles vaccinations | 3001 | -0.7 (-3·6, 2·2) | 0·615 | -3·9% |
| DPT vaccinations | 2576 | -2·1 (-8·2, 4·1) | 0·510 | -7·7% |
| Family planning visits | 2450 | 7·6 (-7·1, 22·3) | 0·307 | 15·6% |
| **Non-targeted aspects of targeted services per month** |  |  |  |  |
| ANC visits | 3179 | 7·2 (-2·7, 17·1) | 0·150 | 19·4% |
| ANC first visits | 3526 | 4·1 (0·01, 8·2) | 0·049 | 23·0% |
| **Non-targeted Services** |  |  |  |  |
| Outpatient visits > 5 yrs | 3353 | -15·8 (-101·1, 69·5) | 0·714 | -4·4% |
| Outpatient visits > 5 yrs, disp. | 2538 | -90·8 (-156·5, -25·2) | 0·007 | -32·8% |
| Outpatient visits < 5 yrs | 3247 | -41·4 (-93·2, 10·9) | 0·120 | -18·4% |
| Outpatient visits < 5 yrs, disp. | 2428 | -57·5 (-110·2, -4·9) | 0·033 | -34·9% |

Note: Standard errors used to calculate confidence intervals are adjusted for clustering at the facility level. N is the number of observations (facility-months).
