# Supplementary material for: Effect of Paying for Performance on Utilisation, Quality, and User Costs of Health Services in Tanzania: A Controlled Before and After Study
Source: PLoS One. 2015 Aug 28;10(8):e0135013. doi: 10.1371/journal.pone.0135013 (PMC4552688; doi:10.1371/journal.pone.0135013)
Supplement: S3 File — (DOCX) [file pone.0135013.s003.docx]

**S3 File: Comparison of baseline characteristics**

**Table A: Comparison of facility characteristics in intervention and comparison areas at baseline**

|  | Intervention (n=75) | Comparison (n=73) | Difference | P-value |
| --- | --- | --- | --- | --- |
| **Ownership** |  |  |  |  |
| Government (%) | 82·7 | 82·2 | 0·5 | 0·940 |
| Faith-based (%) | 12·0 | 13·7 | -1·7 | 0·759 |
| Military/parastatal (%) | 5·3 | 4·1 | 1·2 | 0·728 |
| **Staff/Beds** |  |  |  |  |
| Clinicians, mean [sd] | 3·1 [5·9] | 2·7 [4·7] | 0·4 | 0·622 |
| Nurses, mean [sd] | 6·0 [17·5] | 4·1 [9·4] | 1·9 | 0·422 |
| Paramedics, mean [sd] | 4·7 [10·0] | 4·4 [8·6] | 0·3 | 0·861 |
| Beds, mean [sd] | 3·9 [5·3] | 2·8 [3·6] | 1·1 | 0·168 |
| **Utilities** |  |  |  |  |
| Electricity (%) | 69·0 | 70·1 | -1·1 | 0·886 |
| Water (%) | 73·3 | 79·5 | -6·1 | 0·385 |
| **Services** |  |  |  |  |
| 24hr delivery care (%) | 85·9 | 93·0 | -7·0 | 0·175 |

*CHF: Community Health Fund

**Table B: Comparison of characteristics of available facility sample: outpatient under five for dispensaries**

|  | Intervention (n=27) | Comparison (n=42) | Difference | P-value |
| --- | --- | --- | --- | --- |
| **Ownership** |  |  |  |  |
| Government (%) | 88·9 | 85·4 | 3·5 | 0·680 |
| Faith-based (%) | 7.4 | 14.6 | -7.2 | 0·373 |
| Military/parastatal (%) | 3.7 | 0.0 | 3.7 | 0·220 |
| **Staff/Beds** |  |  |  |  |
| Clinicians, mean [sd] | 1·6 [3.4] | 0·9 [0.8] | 0·6 | 0·260 |
| Nurses, mean [sd] | 3·1 [6.6] | 1·4 [1.6] | 1·8 | 0·104 |
| Paramedics, mean [sd] | 1·5 [1.8] | 1·2 [1.1] | 0·3 | 0·369 |
| Beds, mean [sd] | 1·9 [1.7] | 2·5 [2.7] | -0·6 | 0·327 |
| **Utilities** |  |  |  |  |
| Electricity (%) | 52·2 | 60·5 | -8·4 | 0·531 |
| Water (%) | 66·7 | 70·7 | -4·1 | 0·727 |
| **Services** |  |  |  |  |
| 24hr delivery care (%) | 78·3 | 97·5 | -19·0 | 0·012 |

**Table C: Household characteristics at baseline**

|  | Intervention  (n=1,393) | Comparison  (n=1,489) | Difference | P-value |
| --- | --- | --- | --- | --- |
| **Mother’s characteristics** |  |  |  |  |
| Catholic (%) | 7·0 | 23·5 | -16·5 | 0·000 |
| Protestant (%) | 5·8 | 9·3 | -3·5 | 0·036 |
| Muslim (%) | 86·4 | 66·8 | 19·6 | 0·000 |
| Other religion (%) | 0·70 | 0·40 | 0·3 | 0·485 |
| Married (%) | 70·1 | 63·9 | 6·2 | 0·014 |
| Age in years, mean [sd] | 26·4 [6·7] | 26·2 [6·5] | 0·2 | 0·462 |
| No education (%) | 19·8 | 19·9 | -0·1 | 0·949 |
| Some primary education (%) | 8·6 | 7·7 | 0·9 | 0·434 |
| Primary / some secondary (%) | 65·9 | 63·9 | 2·0 | 0·396 |
| Secondary or above (%) | 5·7 | 8·5 | -2·8 | 0·040 |
| Formal sector job (%) | 1·8 | 2·0 | -0·2 | 0·663 |
| Farmer (%) | 45·9 | 54·6 | -8·7 | 0·043 |
| Self-employed business (%) | 22·0 | 19·7 | 2·3 | 0·314 |
| Taking care of child at home (%) | 28·9 | 22·1 | 6·8 | 0·015 |
| No occupation (%) | 1·3 | 1·6 | -0·3 | 0·560 |
| Number of pregnancies mean [sd] | 2·7 [1·8] | 2·6 [1·7] | 0·1 | 0·546 |
| **Child characteristics** |  |  |  |  |
| Age in months mean [sd] | 7·8 [3·0] | 8·5 [2·9] | -0·7 | 0·000 |
| **Household characteristics** |  |  |  |  |
| Health insurance (%) | 8·6 | 8·5 | 0·1 | 0·962 |
| Household members, mean [sd] | 4·8 [1·8] | 4·9 [1·8] | -0·1 | 0·163 |
| Household wealth (index), mean [sd] | -0·4 [2·8] | 0·3 [3·3] | -0·7 | 0·030 |
| Household head has no education (%) | 2·1 | 1·4 | 0·7 | 0·267 |
| Household head has some primary education (%) | 23·2 | 22·3 | 0·9 | 0·664 |
| Household head has primary or some secondary education (%) | 67·1 | 66·0 | 1·1 | 0·598 |
| Household head has secondary education or above (%) | 7·5 | 10·3 | -2·8 | 0·074 |

**Table D: Characteristics of patients attending facilities (exit survey)**

|  | Intervention  (n=700) | Comparison  (n=762) | Difference | P-value |
| --- | --- | --- | --- | --- |
| **Mother’s characteristics** |  |  |  |  |
| Age of woman, mean [sd] | 26·6 [6·8] | 27·0 [7·1] | -0·5 | 0·222 |
| Insured (%) | 10·1 | 8·1 | 2·0 | 0·354 |
| No education (%) | 26·7 | 26·6 | 0·1 | 0·971 |
| Primary education (%) | 64·3 | 62·9 | 1·4 | 0·635 |
| Secondary education (%) | 8·9 | 9·6 | -0·7 | 0·705 |
| Above secondary (%) | 0·1 | 1·0 | -0·9 | 0·017 |
| Formal employment (%) | 3·3 | 4·7 | -1·4 | 0·216 |
| Informal employment (%) | 71·8 | 75·6 | -3·8 | 0·276 |
| No employment (%) | 24·9 | 19·6 | 5·3 | 0·068 |
| Catholic (%) | 9·8 | 23·6 | -13·8 | 0·000 |
| Protestant (%) | 6·4 | 11·5 | -5·1 | 0·008 |
| Muslim (%) | 82·9 | 64·6 | 18·3 | 0·000 |
| Other religion (%) | 0·9 | 0·3 | 0·6 | 0·251 |
| Married (%) | 69·0 | 67·1 | 1·9 | 0·534 |
| **Child characteristics** |  |  |  |  |
| Age in months mean [sd] | 15·1[13·1] | 15·4[13·4] | -0·3 | 0·714 |
| Child male (%) | 43·7 | 47·9 | -4·3 | 0·222 |
| **Household characteristics** |  |  |  |  |
| Household members, mean [sd] | 6·3 [3·3] | 5·6 [2·7] | 0·7 | 0·002 |
| Household wealth (index), mean [sd] | -0·07 [3·1] | 0·06[3·6] | -0·13 | 0·733 |
| Household head has some primary education (%) | 83·0 | 85·3 | -2·3 | 0·382 |
| Household head has primary or some secondary education (%) | 9·0 | 8·6 | 0·4 | 0·852 |
| Household head has secondary education or above (%) | 2·2 | 3·4 | -1·2 | 0·235 |
