# Supplementary material for: Effect of Paying for Performance on Utilisation, Quality, and User Costs of Health Services in Tanzania: A Controlled Before and After Study
Source: PLoS One. 2015 Aug 28;10(8):e0135013. doi: 10.1371/journal.pone.0135013 (PMC4552688; doi:10.1371/journal.pone.0135013)
Supplement: S4 File — (DOCX) [file pone.0135013.s004.docx]

**S4 File: Results using linear model with standard errors clustered at the district level, adjusting for limited number of clusters using the wild cluster bootstrap approach outlined in Cameron et al. (2008)**

We present the estimated standard errors for those outcomes which were statistically significant in the models with clustering at facility level.

**Table A: Direct and indirect effect of P4P on the use of targeted services**

|  | Baseline survey | | | | Difference in difference, effect | | | |
| --- | --- | --- | --- | --- | --- | --- | --- | --- |
|  | **Intervention** | **Comparison** | **Difference** | **P-value** | **N** | **Beta† (95% CI)** | **P-Value** | **%D*** |
| **Targeted services** |  |  |  |  |  |  |  |  |
| At least 2 doses of IPT during ANC (%) | 49·5 | 56·7 | -7·2 | 0·005 | 4759 | 10.3 [5.8 to 15.0] | 0·005 | 20·8 |
| Institutional delivery rate (%) | 84·7 | 86·8 | -2·1 | 0·350 | 5747 | 8.2 [0.9 to 14.7] | 0·095 | 9·7 |
| Institutional delivery rate (public) (%) | 76·8 | 77·8 | -1·0 | 0·786 | 5747 | 6.5 [1.1 to 11.2] | 0·055 | 8·5 |
| Polio vaccine at birth (%) | 77·4 | 78·5 | -1·1 | 0·668 | 5747 | 5.6 [-2.7 to 13.7] | 0·255 | 7·2 |
| **Non-targeted aspects of targeted services** |  |  |  |  |  |  |  |  |
| Any ANC visit (%) | 97·2 | 99·9 | -2·7 | 0·001 | 5742 | 3.3 [2.2 to 4.4] | 0·000 | 3·4 |

*The % D = (beta / baseline mean) × 100, where the baseline mean of the dependent variable is for the intervention group. †The Beta is the estimated intervention effect controlling for a year dummy, facility-fixed effects, individual-level and household characteristics. ^Among infants aged 6-11 months.

**Table B: Effect of P4P on the use of non-targeted services**

|  | Baseline survey | | | |  | Difference in difference, effect | | | |
| --- | --- | --- | --- | --- | --- | --- | --- | --- | --- |
|  | **Intervention** | **Comparison** | **Difference** | **P-value** | **Facilities** | **N** | **Beta† (95% CI)** | **P-Value** | **%D*** |
| Outpatient visits per month > 5 yrs. | 359·5 | 287·3 | 72·2 | <0·001 | 96 | 3353 | -15.8 [-84.6 to 53.9] | 0.735 | -4.4% |
| Outpatient visits per month > 5 yrs, dispensaries | 276·8 | 235·4 | 41·4 | 0·006 | 69 | 2538 | -90·8 [-150.7 to -24.5] | 0·035 | -32·8% |
| Outpatient visits per month < 5 yrs. | 223·9 | 193·7 | 30·2 | 0·011 | 93 | 3247 | -41.1 [-64.3 to -18.1] | 0.035 | -18.4% |
| Outpatient visits per month < 5 yrs, dispensaries | 164·8 | 172·6 | -7·8 | 0·441 | 72 | 2428 | -57·5 [-93.3 to -20.9] | 0·010 | -34·9% |

Note to Table: N=facility months; *The % D = (beta / baseline mean) × 100, where the baseline mean of the dependent variable is for the intervention group. †The Beta is the estimated intervention effect controlling for a year dummy and facility-fixed effects.

**Table C: Effect of P4P on quality of care**

|  | Baseline survey | | | | Difference-in-difference, effect | | | |
| --- | --- | --- | --- | --- | --- | --- | --- | --- |
|  | **Intervention mean [sd]** | **Comparison mean [sd]** | **Difference** | **P-value** | **N** | **Beta† [95% CI]** | **P-Value** | **%D*** |
| **Targeted services** |  |  |  |  |  |  |  |  |
| Patient assessment of staff kindness during delivery score (1-10)^ | 7·2 [2·7] | 7·6 [2·7] | -0·4 | 0·009 | 4920 | 0·38 [0.08 to 0·67] | 0·07 | 5·3 |
| **Non-targeted services** |  |  |  |  |  |  |  |  |
| Index of patient satisfaction with interpersonal care | 0·69 [0·18] | 0·74 [0·15] | -0·05 | 0·007 | 1170 | 0·05 [0·03 to 0·07] | 0·00 | 7·2 |

Note to Table: Same sizes as indicated at top except where indicated ^. ^ Data from household survey: sample size; *The % D = (beta / baseline mean) × 100, where the baseline mean of the dependent variable is for the intervention group. †The Beta is the estimated intervention effect controlling for a year dummy, facility-fixed effects, individual-level and household characteristics

**Table D: Effect of P4P on the probability of paying for services in public facilities**

|  | Baseline survey | | | | Difference in difference, effect | | | |
| --- | --- | --- | --- | --- | --- | --- | --- | --- |
| Service cost | **Intervention** | **Comparison** | **Difference** | **P-value** | **N** | **Beta† [95% CI]** | **P-Value** | **%D*** |
| Prob. of paying Delivery (%) | 16.5 | 11.9 | 4.6 | 0·026 | 4485 | -5.0 [-7.2 to -2.8] | 0·010 | -30·3 |

*The % D = (beta / baseline mean) × 100, where the baseline mean of the dependent variable is for the intervention group. †The Beta is the estimated intervention effect controlling for a year dummy, facility-fixed effects, individual-level and household characteristics

**Table E: Equity effects of P4P**

|  | N | P4P effect among: | |
| --- | --- | --- | --- |
| Variables |  | Poorest group β (P-value) | Middle group β (P-value) |
| Institutional delivery rate | 5747 | 6.2 (0·355) | 7.0 (0·18) |
| Institutional delivery rate (public facilities) | 5747 | 10.5 (0·145) | 3.8 (0·49) |
| Prob. Of paying for delivery care | 4485 | -8.9 (0·13) | -7.2 (0·185) |

Source: Household survey.

Note: Richest wealth group=reference group; Covariates were marital status, health insurance, education, occupation, religion, parity, age, wealth terciles and household size.
