# Supplementary material for: Effect of Paying for Performance on Utilisation, Quality, and User Costs of Health Services in Tanzania: A Controlled Before and After Study
Source: PLoS One. 2015 Aug 28;10(8):e0135013. doi: 10.1371/journal.pone.0135013 (PMC4552688; doi:10.1371/journal.pone.0135013)
Supplement: S5 File — (DOCX) [file pone.0135013.s005.docx]

**S5 File: Results using logit model on all binary outcomes (with marginal effects and standard errors clustered at the facility level)**

**Table A: Direct and indirect effect of P4P on the use of targeted services**

|  | Baseline survey | | | | Difference in difference, effect | | |
| --- | --- | --- | --- | --- | --- | --- | --- |
|  | **Intervention** | **Comparison** | **Difference** | **P-value** | **N** | **dy/dx (95% CI)** | **P-Value** |
| **Targeted Services** |  |  |  |  |  |  |  |
| At least 2 doses of IPT during ANC (%) | 49·5 | 56·7 | -7·2 | 0·005 | 4759 | 9.7 (3.9, 15.5) | 0·001 |
| HIV treatment during ANC (%) | 7·8 | 6·8 | 1·0 | 0·527 | 4453 | -0.4 (-5.4, 4.5) | 0.864 |
| Institutional delivery rate (%) | 84·7 | 86·8 | -2·1 | 0·350 | 5280 | 9.6 (4.8, 14.3) | 0·000 |
| Institutional delivery rate (public) (%) | 76·8 | 77·8 | -1·0 | 0·786 | 5479 | 7.1 (1.8, 12.3) | 0·008 |
| Polio vaccine at birth (%) | 77·4 | 78·5 | -1·1 | 0·668 | 5747 | 5.6 (-0.9, 12.1) | 0·091 |
| Measles (%) | 51·4 | 53·3 | -1·9 | 0·654 | 1225 | 8.8 (-3.5, 21.1) | 0·159 |
| Penta 3 doses^ (%) | 76·4 | 79·9 | -3·5 | 0·243 | 2338 | 2.5 (-7.2, 12.2) | 0·616 |
| Postnatal care in facility<7 days (%) | 21·5 | 16·9 | 4·6 | 0·043 | 5671 | 1.3 (-4.3, 6.8) | 0·659 |
| Use of any family planning (%) | 36·7 | 39·2 | -2·5 | 0·398 | 5495 | -1.0 (-7.6, 5.6) | 0·766 |
| **Non-targeted aspects of targeted services** |  |  |  |  |  |  |  |
| Any ANC visit (%) | 97·2 | 99·9 | -2·7 | 0·001 | 1104 | 23.4 (11.8, 35.1) | 0·000 |
| Four or more ANC visits (%) | 65·0 | 71·2 | -6·2 | 0·020 | 5674 | 4.1 (-2.4, 10.5) | 0·219 |
| Postnatal care in facility < 2 months (%) | 27·7 | 23·4 | 4·3 | 0·120 | 5745 | -0.9 (-7.3, 5.5) | 0·776 |

**Table B: Effect of P4P on the probability of paying for services in a public facility or offering a gift**

|  | Baseline survey | | | | Difference in difference, effect | | |
| --- | --- | --- | --- | --- | --- | --- | --- |
| **Service cost** | **Intervention** | **Comparison** | **Difference** | **P-value** | **N** | **dy/dx [95% CI]** | **P-Value** |
| Prob. of paying ANC (%) | 8.1 | 7.5 | 0.6 | 0·711 | 4139 | -3.6 [-7.7, 0.4] | 0·081 |
| Prob. of paying Delivery (%) | 16.5 | 11.9 | 4.6 | 0·026 | 4094 | -4.9 [-9.7, -0·0] | 0·048 |
| Prob. of paying PNC (%) | 6·0 | 7·6 | -1·6 | 0·421 | 521 | 4.0 [-10.4, 18.4] | 0·584 |
| Provided a gift for ANC (%) | 1·7 | 1·2 | 0·5 | 0·403 | 1942 | 3.5 [-0.6, 7.6] | 0·093 |
| Provided a gift for delivery (%) | 17·4 | 18·8 | -1·4 | 0·586 | 4243 | -3.0 [-8.4, 2.4] | 0·270 |
| Provided a gift for PNC (%) | 7·1 | 4·5 | 2.6 | 0·186 | 582 | 0.4 [-12.9, 13.8] | 0·950 |

**Table C: Equity effects of P4P**

|  | N | P4P effect among: | |
| --- | --- | --- | --- |
| Variables |  | Poorest group  **dy/dx** (P-value) | Middle group  **dy/dx** (P-value) |
| At least 2 doses of IPT during ANC | 4759 | 10.3 (0·142) | 10.6 (0·110) |
| Institutional delivery rate | 5280 | 0.8 (0·889) | 2.1 (0·696) |
| Institutional delivery rate (public facilities) | 5479 | 9.8 (0·093) | 3.6 (0·484) |
| Polio vaccine at birth | 5747 | 0·3 (0·959) | 2.0 (0·740) |
| Prob. of paying for delivery care | 4094 | -7.9 (0·175) | -7.7 (0·159) |

Source: Household survey.

Note: Richest wealth group=reference group; Covariates were marital status, health insurance, education, occupation, religion, parity, age, wealth terciles and household size.
