# Supplementary material for: Effect of Paying for Performance on Utilisation, Quality, and User Costs of Health Services in Tanzania: A Controlled Before and After Study
Source: PLoS One. 2015 Aug 28;10(8):e0135013. doi: 10.1371/journal.pone.0135013 (PMC4552688; doi:10.1371/journal.pone.0135013)
Supplement: S1 Table — (DOCX) [file pone.0135013.s006.docx]

**S1 Table: Overview of core indicators for each of the surveys**

| **Type of Impact** | **Indicators** | **Data Source** | **Pre-specified (Y/N)** | **Incent-ivised (Y/N)** |
| --- | --- | --- | --- | --- |
| Quality of care | Average waiting time for targeted services in minutes | Exit interview | Y | N |
|  | Average waiting time non-targeted services in mins | Exit interview | Y | N |
|  | Average consultation time for targeted services in mins | Exit interview | Y | N |
|  | Average consultation time for non targeted services in mins | Exit interview | Y | N |
|  | % prescribed drugs for malaria (two doses) during ANC | Household survey | Y | Y |
|  | % treated for HIV during ANC | Household survey | Y | Y |
|  | Patient satisfaction with inter-personal care for targeted services measured as an index | Exit interview | N | N |
|  | Patient satisfaction with inter-personal care for non-targeted services measured as an index | Exit interview | N | N |
| Service utilisation | % women delivering in a health facility | Household survey | Y | Y |
|  | % of women having any ANC | Household survey | N | N |
|  | % of women who had 4 or more ANC visits | Household survey | Y | N |
|  | % women who received postnatal care within 7 days of birth in a health facility | Household survey | N | Y |
|  | % women who received postnatal care within 2 months of birth in a health facility | Household survey | Y | N |
|  | % of children receiving polio at birth | Household survey | Y | Y |
|  | % of children receiving Penta 3 (among appropriate age group) | Household survey | Y | Y |
|  | % measles fully immunised for measles (among appropriate age group) | Household survey | Y | Y |
|  | % women currently using a family planning method | Household survey | Y | Y |
|  | Mean annual outpatient visits under 5 | Facility survey | Y | N |
|  | Mean annual outpatient visits over 5 years | Facility survey | Y | N |
|  | Mean annual outpatient visits under 5 in dispensaries | Facility survey | N | N |
|  | Mean annual outpatient visits over 5 years in dispensaries | Facility survey | N | N |
|  | Mean annual ANC service utilisation (all ANC and first ANC) | Facility survey | Y | N |
|  | Mean annual delivery service utilisation (normal delivery) | Facility survey | Y | N |
|  | Mean annual FP visits | Facility survey | Y | Y |
|  | Mean number of under 1 year olds receiving Penta 3 vaccine | Facility survey | Y | Y |
|  | Mean number of under 1 year olds receiving polio vaccine | Facility survey | Y | N |
|  | Mean number of under 1 year olds receiving measles vaccine | Facility survey | Y | Y |
| Economic effects | % paying for delivery at public facility | Household survey | Y | N |
|  | % paying ANC at public facility | Household survey | N | N |
|  | % paying for PNC at public facility | Household survey | N | N |
|  | Amount paid for delivery care at public facility | Household survey | N | N |
|  | Amount paid for ANC at public facility | Household survey | N | N |
|  | Amount paid for PNC at public facility | Household survey | N | N |
|  | % offering gift for delivery at public facility | Household survey | N | N |
|  | % offering gift for ANC at public facility | Household survey | N | N |
|  | % offering gift for PNC at public facility | Household survey | N | N |
| Equity | % service use among poorest compared to least poor women/children for all significant outcomes | Household survey | Y | N |
|  | % reporting payment for services among poorest compared to least poor women | Household survey | Y | N |
