# Supplementary material for: Effect of Paying for Performance on Utilisation, Quality, and User Costs of Health Services in Tanzania: A Controlled Before and After Study
Source: PLoS One. 2015 Aug 28;10(8):e0135013. doi: 10.1371/journal.pone.0135013 (PMC4552688; doi:10.1371/journal.pone.0135013)
Supplement: S2 Table — (DOCX) [file pone.0135013.s007.docx]

**S2 Table: Equity effects of P4P**

|  | N | P4P effect among | |
| --- | --- | --- | --- |
| Variables |  | Poorest group  β (P-value) | Middle group  β (P-value) |
| Service use |  |  |  |
| At least 2 doses of IPT during ANC | 4759 | 10.1 (0·152) | 9.6 (0·156) |
| Institutional delivery rate | 5747 | 6.2 (0·214) | 7.0 (0·080) |
| Institutional delivery rate (public facilities) | 5747 | 10.5 (0·053) | 3.8 (0·419) |
| Polio vaccine at birth | 5747 | 1.8 (0·779) | 2.8 (0·627) |
| Quality of care |  |  |  |
| Kindness mean ranks | 4920 | -0·37 (0·388) | 0·17 (0·669) |
| Prob. of paying for delivery care | 4485 | -8.9 (0·087) | -7.2 (0·138) |

Source: Household survey

Note: Richest wealth group=reference group; Covariates were marital status, health insurance, education, occupation, religion, parity, age, wealth index and household size.
